# CHRFAM7A variants regulate the neuronal cytoskeleton through distinct mechanisms

**DOI:** 10.1002/alz70861_108926

**Published:** 2025-12-23

**Authors:** Nicolas Rosas, Ivanna Ihnatovych, Kinga Szigeti

**Affiliations:** ^1^ University at Buffalo, Buffalo, NY USA

## Abstract

**Background:**

Human‐specific genes play a crucial role in the structural and functional differences of the human brain, making it distinct from other species. *CHRFAM7A* is a human specific gene associated with Alzheimer’s disease originated from the fusion between the alpha 7 nicotinic acetylcholine receptor (α7nAChR) and ULK4, a member of the ULK kinase family. *CHRFAM7A* has three alleles: the ancestral allele lacking the fusion gene (0 copy, rare), the fusion gene in direct orientation (*CHRFAM7A, 50%*), and the fusion gene in inverted orientation characterized by a 2bp deletion in exon 6 (CHRFAM7AΔ2bp, 50%). Translated CHRFAM7A from the direct allele modifies the activity of α7nAChR while the function of the inverted allele is unknown. Based on our previous work eluding to a role in cytoskeletal function we pursued characterization of the alleles to uncover the mechanism for disease association.

**Method:**

Isogenic iPSC derived medial ganglionic eminence (MGE) neuronal progenitors are studied by live imaging using probes LifeActin‐RFP for actin cytoskeleton or SPY555‐tubulin for microtubules. ImageJ was used to analyze debundling, neurite movement and length, branching and type of process. ULK4 siRNA knockdown was utilized for pathway exploration.

**Result:**

Phenotypically direct MGE progenitors have increased growth cone movement with lamellipodia membrane specialization. In neurons, CHRFAM7A shifts dendritic spine type from filopodia to long thin spines. In contrast, *CHRFAM7AΔ2bp* MGE progenitors demonstrate an increase in neurite tip exploratory movement, higher arborization and debundling of the neuronal processes. *CHRFAM7AΔ2bp* affects ULK4 expression and a‐tubulin acetylation of the microtubule cytoskeleton.

**Conclusion:**

*CHRFAM7A* alleles have distinct molecular mechanisms. Translated CHRFAM7A from the direct allele regulates the actin cytoskeleton while CHRFAM7A_Δ2bp affects microtubule remodeling through ULK4. These variants could differentially affect neuronal adaptation to changes in the brain environment in Alzheimer's disease.

